# Cardiac Autonomic Reactivity Tests in Type A and Type B Personalities

**DOI:** 10.7759/cureus.53690

**Published:** 2024-02-06

**Authors:** Imtiyaz Bharti, Waqas Alauddin, Prajakta M Radke, Anant Patil, Rayyan Sunasra, Adnan Acharwala

**Affiliations:** 1 Department of Physiology, Rama Medical College, Ghaziabad, IND; 2 Department of Physiology, Naraina Medical College & Research Centre, Kanpur, IND; 3 Department of Physiology, Dr. D. Y. Patil Medical College, Navi Mumbai, IND; 4 Department of Pharmacology, Dr. D. Y. Patil Medical College, Navi Mumbai, IND; 5 College of Medicine, Hinduhridaysamrat Balasaheb Thackeray Medical College & Dr. R.N. Cooper Municipal General Hospital, Mumbai, IND

**Keywords:** type a and b personalities, cardiac autonomic functions, parasympathetic tone, sympathetic tone, autonomic function tests

## Abstract

Background: A considerable link between personality types and cardiovascular diseases (CVDs) has been seen. Autonomic responses in both type A and type B personality individuals were found to be influenced by their personality traits. The study suggests further research on cardiac autonomic functions in larger sample sizes and the use of non-invasive screening techniques like cardiovascular reflex tests to stratify participants' risk of future illness.

Objective: This study aimed to assess autonomic stress reactivity tests in type A and type B personalities using cardiovascular reflex tests.

Methods: This study was conducted at the Department of Physiology, Vardhman Mahavir Medical College and Safdarjung Hospital, New Delhi. The Hunter-Wolf Personality Questionnaire Scale was used to identify 60 adults, 30 of whom were classified to have type A personality and 30 have type B personality, from the psychiatry department. Autonomic function tests, such as the handgrip tests, cold pressor test, deep breathing test (DBT), lying-to-standing test (LST), and Valsalva maneuver, were performed and recorded for each subject. IBM SPSS Statistics for Windows, version 21 (released 2012; IBM Corp., Armonk, New York, United States) was used for the compilation and analysis of data.

Results: The E:I (expiration-to-inspiration) ratio and delta heart rate of the type A personality patients both significantly decreased (p = 0.000*) as compared to the type B personality patients (1.18 ± 0.03 versus 1.25 ± 0.77 and 1.18 ± 0.03 versus 1.25 ± 0.77). The Valsalva ratio of the type A personality patients decreased (1.38 ± 0.10) as compared to the type B personality patients (1.48 ± 0.18), which was statistically significant (p = 0.001*). The 30:15 ratio in the type A personality patients was significantly decreased (p = 0.03*) compared to the type B personality patients (1.12 ± 0.05 versus 1.15 ± 0.10). The handgrip test and cold pressor test results were statistically insignificant.

Conclusion: Compared to the type B personality patients, which exhibited an increase in both parasympathetic and sympathetic reactivity, the type A personality patients exhibited a reduction in resting cardiovascular parameters and resting autonomic tone. Consequently, in order to stratify the participants' risk of future illness, we recommend employing non-invasive procedures, such as cardiovascular reflex tests, as a screening technique.

## Introduction

Cardiovascular diseases (CVDs) are now a global burden, accounting for one-third of all fatalities worldwide [1]. Knowing the early risk factors for CVDs is crucial because of this high burden. By doing so, preventative measures can be targeted and guided, or strategies that encourage lifelong healthy behaviors and attitudes can be adopted [2]. The first to recognize and define a link between personality and CVDs were Friedman and Rosenman [3]. The two cardiologists described type A behavior in 1959 as an action-emotion complex that is evident in anyone actively engaged in a persistent, relentless attempt to accomplish several goals at the same time [4]. Specifically, the type A personality is marked by a strong drive to accomplish goals, setting strict deadlines for oneself, completing tasks quickly, deliberately participating in a variety of activities or leadership roles, and displaying competitive or aggressive behavior. This type of behavior is also called the coronary-prone behavior [2]. The absence of these type A personality attributes identifies type B personality. Numerous studies have found a direct correlation between cardiovascular illness and type A personalities [5]. Research has shown that intense psychosocial feelings, like violence or situational rage, together with competitive thinking, are associated with increased hemodynamic reactivity and increase the risk of myocardial infarction in the near term [6].

Lee and Watanuki's study of personality types and autonomic responses discovered a link between autonomic responses and autonomic measures [7]. Because of increased sympathetic activity, both type A and type B personalities showed a lower parasympathetic tone, with type A displaying a higher sympathetic reactivity [7].

Furthermore, given that type A and B personalities can have varying effects on CVDs throughout life, there is a dearth of information on the likely associations between these traits and cardiovascular risk factors in childhood [2]. Understanding the potential links between personality types and cardiovascular risk factors in childhood is crucial due to the potential long-term impact on cardiovascular health.

This study aims to analyze type A and B personalities and autonomic stress reactivity in adults, providing insights into how personality traits influence physiological responses to stress. To the best of our knowledge, this is the first study to analyze these interactions, aiming to fill a gap in knowledge and shed light on the complex interplay between the personality type and cardiovascular autonomic reactivity in adults.

## Materials and methods

This cross-sectional study was conducted from November 2019 to April 2021 at the Department of Physiology, Vardhman Mahavir Medical College and Safdarjung Hospital, New Delhi. Before starting, each participant had given written consent. The Hunter-Wolf Personality Questionnaire Type A and B scales were used to assess 60 healthy young adults with type A and B personalities; 30 people between the ages of 18 and 25 were in each group [2]. Two participants were asked to respond to 24 questions. Every question had two polar responses. The participants were asked to choose the closest number from one to seven to their responses. The numbers from all of the solutions have been added together. Following that, the personalities were classified based on their overall score. Type A personality ranges from 120 to 168 total points, while type AB personality ranges from 85 to 119 total points and type B personality ranges from 24 to 84 total points. Autonomic function was tested in people with type A and type B personalities. Patients with autoimmune diseases, hepatorenal, endocrine disorders, hypertension, diabetes mellitus, type AB personality, heart failure, cardiac disease, neuropsychiatric disorders, drug addiction, and other medical comorbidities were not allowed to participate in this study.

Study design

Methodology for Evaluating Cardiac Autonomic Stress Reactivity Examinations

According to conventional protocols described in the literature, a deep breathing test, lying-to-standing test, Valsalva maneuver, cold pressor test, and hand grip test were carried out [8]. The subjects were told to abstain from food and caffeine for four hours prior to the start of the experiment. Throughout the examinations, brachial artery blood pressure and lead II ECG were recorded.

Deep breathing examination: The deep breathing test evaluates cardiac parasympathetic activity. The vagal nerve mediates cardiac reactions to deep breathing; hence, this is referred to as a cardiovagal test. ECG records are used to determine heart rate (HR) changes. For 30 seconds, an ECG baseline is recorded. The individual is then instructed for deep inhalation followed by a deep expiration, with each of the breathing cycle lasting 10 seconds. Six cycles have been documented. During the maneuver, ECG traces are used to compute inspiratory and expiratory HRs and RR intervals for each of the six cycles.

Delta HR is the dissimilarity between the highest and lowest HRs during inspiration and expiration, an average of over six cycles. E:I (expiration-to-inspiration) ratio is the average of the longest and shortest RR intervals across six cycles.

Valsalva maneuver: Cardiovagal, i.e., parasympathetic, functioning is assessed using HR responses during the Valsalva maneuver. The subject is asked to blow into a mouthpiece attached to a sphygmomanometer for 15 seconds in order to maintain an expiratory pressure of 40 mmHg. Deep breathing is avoided both before and soon after the maneuver.

The Valsalva ratio is calculated as the longest RR interval during phase IV divided by the shortest RR interval during phase II.

Handgrip examination: The diastolic blood pressure (DBP) responses to measurable isometric exercise stimuli are used to examine sympathetic adrenergic functioning in the handgrip test. After taking the subject's initial blood pressure and demonstrating how to use the handgrip dynamometer, the test is explained to the subject, and the person is given directions to hold the dominant hand as forcefully as possible for a few seconds. The result is recorded, and the process is repeated three times. The maximum value of the three readings is their maximal voluntary contraction (MVC). The dynamometer is marked at 30% of the subject's MVC. An isometric contraction using a handgrip dynamometer at 30 percent of the maximum voluntary contraction is required of the subject for four minutes. The blood pressure in the opposing arm is taken during the test. There is an increase in diastolic pressure over the reference value.

Delta diastolic BP is the greatest DBP during the test minus the baseline DBP.

Cold pressor examination: The cold pressor test uses DBP responses to cold stimuli to assess sympathetic adrenergic functioning. One measures their baseline blood pressure. The participant is instructed to submerge their right hand up to the wrist in cold water (10 degrees Celsius) for a duration of one minute. The contralateral arm is used the entire time to take the blood pressure. It is noted when the diastolic pressure rises above baseline.

The greatest DBP during the test less the baseline DBP is the delta diastolic blood pressure.

Lying-to-standing test: The autonomic nervous system's two limbs are assessed by the lying-to-standing test, and the parasympathetic limb is measured by the 30:15 ratio. The ratio of the longest RR interval at or around the 15th beat to the largest RR interval at or around the 30th beat is known as the 30:15 ratio.

Statistical analysis

IBM SPSS Statistics for Windows, version 21 (released 2012; IBM Corp., Armonk, New York, United States), a licensed statistical program, was used to compile, enter, and analyze the data. The mean ± SD was used to express the values. An unpaired t-test was used to determine the statistical significance of the differences between type A and type B personalities. Pearson's coefficient of correlation and regression analysis were used for multivariate analysis. A significance threshold of P < 0.05 was used.

Permission to conduct the study was taken from the Institutional Ethics Review Committee of Vardhaman Mahavir Medical College and Safdarjung Hospital, New Delhi, India (Institutional Ethics Review Committee (IERC) approval number: IEC/VMMC/SJH/Thesis/2019-10/259).

## Results

Sixty healthy individuals with type A and B personalities, ranging in age from 18 to 25, were included in this study. The mean HR of type A personality patients was 84.03 ± 3.38 bpm, whereas that of type B personality patients was 79.56 ± 3.77 bpm (p = 0.000*) as depicted in Table 1. The systolic BP of the type A personality patients was 118.9 ± 4.44 mmHg, whereas that of the type B personality patients was 115.63 ± 4.26 mmHg (p = 0.005*). The diastolic BP of the type A personality patients was 76.20 ± 3.89 mmHg, whereas that of the type B personality patients was 75.13 ± 2.95 mmHg (p = 0.23), as shown in Table 1.

**Table 1:** Comparison of basal parameters between type A and type B personality patients Values are expressed as mean ± SD (standard deviation) or number (%). *p-value < 0.05, statistically significant. BMI: body mass index; n: number; BP: blood pressure; NS: not significant

	Type A (Mean ± SD)	Type B (Mean ± SD)	p-value
Age (years)	23.48 ± 1.52	22.79 ± 2.34	NS
BMI	24.30 ± 1.56	25.56 ± 2.41	NS
Mean heart rate	84.03 ± 3.38	79.56 ± 3.77	0.000*
Systolic BP	118.9± 4.44	115.63 ± 4.26	0.005*
Diastolic BP	76.20 ± 3.89	75.13 ± 2.95	0.23

The E:I ratio in the type A personality patients significantly decreased (p = 0.000*) as compared to the type B personality patients (1.18 ± 0.03 vs. 1.25 ± 0.77), and the delta HR also significantly decreased in the type A personality patients (14.38 ± 0.77 bpm), compared to the type B personality patients (15.95 ± 0.80) (p = 0.000*), as depicted in Table 2. The Valsalva ratio of the type A personality patients (1.38 ± 0.10) decreased as compared to that of the type B personality patients (1.48 ± 0.18), which was statistically significant (p = 0.001*), as depicted in Table 2. The 30:15 ratio in type A was decreased compared to that of type B (1.12 ± 0.05 vs. 1.15 ± 0.10), which was statistically significant (p = 0.03*). The handgrip test and cold pressor test results were also statistically insignificant.

**Table 2:** Comparison of the cardiovascular reflex test between type A and type B personalities The values are expressed as mean ± SD (standard deviation). *p-value < 0.05, statistically significant. Delta HR: delta heart rate, bpm: beats per minute, E:I: expiration/inspiration, delta diastolic blood pressure: change in diastolic blood pressure in the isometric handgrip test, delta systolic blood pressure: systolic blood pressure during the lying-to-standing test; n: number; DBT: deep breathing test; LST: lying-to-standing test

	Type A (mean ± SD)	Type B (mean ± SD)	p-value
Delta HR (DBT)	14.38667 ± 0.77	15.95667 ± 0.80	0.000*
E:I ratio (DBT)	1.19 ± 0.03	1.21 ± 0.07	0.000*
Valsalva ratio	1.38 ± 0.10	1.48 ± 0.18	0.001*
30:15 ratio (LST)	1.12 ± 0.05	1.15 ± 0.10	0.03*
Handgrip test	13.03 ± 9.32	11.03 ± 6.50	0.394
Cold pressor test	8.30 ± 8.26	6.93 ± 6.02	0.766

## Discussion

HR and systolic blood pressure are two cardiovascular parameters in this study that were significantly higher in the type A personality participants than in the type B personality participants.

The cardiovascular reflex tests, such as the E:I ratio and delta HR, in the type A personality patients were both significantly decreased as compared to the type B personality patients. These tests are used to access the parasympathetic tone.

Based on the thickness of the intima-media, a precursor to atherosclerotic heart disease, the only other study that has used information about childhood history to determine the etiological influence of personality on cardiovascular risk is the *Cardiovascular Risk in Young Finns Study*. The findings they discovered were intriguing: having a type A personality as a child was a somewhat significant predictor of having thicker intimate media as an adult. For future research on the etiology of CVDs in type A personalities, they suggested using a life-course approach [9]. The negative health effects of this association may be attributed to factors, such as poor adherence to therapy and an unhealthy lifestyle, and a higher chance of mental illnesses, such as anxiety and depression, which have also been linked to an increased risk of coronary heart disease (CHD) and underlying biological mechanisms, such as inflammation or autonomic disorder imbalance. The biological mechanisms underlying this association are not well understood [10]. Adrenaline is released into the bloodstream from the adrenal cortex, and noradrenaline is produced as a neurotransmitter from the postganglionic neuron as a result of sympathetic nervous system activation. Because of these mental demands, adrenaline and norepinephrine were combined and employed as components of autonomic nerve action [11]. Hence, our findings revealed that the type A personality group demonstrated a decrease in parasympathetic tone in all of the aforementioned parasympathetic reactivity tests and also showed sympathoexcitation depicting autonomic dysfunction when compared to the type B group. In addition, we discovered that HR and systolic blood pressure were significantly higher in the type A participants than in the type B participants.

Since the sympathetic-adrenomedullary system and the hypothalamic-pituitary-adrenocortical axis are two systems that function in tandem during stress, changes in sympathetic parameters may be biologically compared to changes in cortisol [12]. As a result, research has also been done on the relationship between cortisol and sympathoexcitation, a substitute indicator of sympathetic reactions. Our literature has investigated the possible relationship between the vagus nerve and type B personality in great detail. Initially, vagal activation promotes improved emotion regulation and increased emotional intelligence by attenuating the cardiovascular arousal responses [13,14,15]. Second, a theoretical foundation for comprehending the function of cardiac vagal reactivity in emotion regulation is provided by Porges' polyvagal theory [16,17]. The nucleus ambiguus and dorsal motor nucleus are two distinct vagal nuclei in the brain stem, according to this idea. The visceral organs, namely, the heart, larynx, and face muscles, that are crucial to emotions and their transmission are the terminus of the more recent branch of the mammalian nervous system, which is governed by the nucleus ambiguus. Similarly, behavioral reactions and changes in emotional states are mediated by the efferent vagal projections. Increased expressivity, more sociability, and a positive emotional style are predicted by the vagal activity, which is often measured by respiratory sinus arrhythmia (RSA) [18,19,20]. These results essentially suggest that increased parasympathetic and maybe decreased sympathetic activity are linked to pleasure, whereas a shift toward sympathetic dominance is linked to melancholy. In our research, type A personalities showed lower results on the parasympathetic reactivity tests (E:I ratio and delta HR values), which indicates a lower parasympathetic tone.

Research has shown that the parasympathetic nervous system's activity is linked to higher-order behavior and cognition [21]. A higher vagal tone is indicative of a more adaptable response to changes in environmental demands [22]. Our study also revealed similar results: the type B group had a better parasympathetic tone, which we have seen in the Valsalva ratio, deep breathing test, and delta HR. The Valsalva ratio was much lower in the type A group than in the type B group, indicating a lower parasympathetic tone. In addition, there was a statistically significant decrease in the 30:15 ratio.

Kamada et al. discovered in their study that there was a significant difference in sympathovagal balance between types A and B, with type A being the dominant sympathetic activity. These results are consistent with our findings. In line with our results, Kamada et al. [23] discovered that type A has the predominant sympathetic activity and that there was a significant difference in the sympathovagal balance between types A and B. Our findings showed that there was increased HR and systolic blood pressure pointing toward increased sympathetic activity and decreased parasympathetic tone causing autonomic dysfunction in type A personality as compared to type B personality.

Regarding this study's limitations, we would advise extensive longitudinal research with a larger sample size to get more definitive findings. The study's patient population was small. It is crucial to do a study that exclusively includes women because the study population only consisted of men. More research should be done in this area because there have been conflicting findings regarding autonomic reactivity in different personality types.

## Conclusions

We draw the preliminary study's conclusion that type A personality's autonomic functions were inferior to those of type B, which demonstrated both baseline autonomic cardiac tone and autonomic reactivity. We suggest more research with a bigger sample size on the autonomic functions of the heart in type A and type B personalities. Consequently, in order to stratify the participants' risk of future illness, we recommend employing non-invasive procedures, such as cardiovascular reflex tests, as a screening technique.
